# *“… I tried to commit suicide…”:* Understanding the intersections between mental health, HIV and teenage pregnancy

**DOI:** 10.1371/journal.pone.0323030

**Published:** 2026-01-05

**Authors:** Hanani Tabana, Takunda Satumba, Diane Cooper, Martina Lembani

**Affiliations:** School of Public Health, University of the Western Cape, Cape Town, South Africa; Sefako Makgatho Health Sciences University, SOUTH AFRICA

## Abstract

**Background:**

Adolescent girls in South Africa face a range of interconnected health challenges, including high rates of unplanned pregnancy and HIV infection, which are compounded by systemic inequities in accessing sufficient health care. These vulnerabilities can significantly affect their mental health and overall well-being. This paper describes adolescent girls’ lived experiences when accessing health care services and how these experiences might be associated with mental health, HIV, and teenage pregnancy in the Western Cape province, South Africa.

**Methods:**

This qualitative study employed narrative and semi-structured interviews to explore the sexual and reproductive health and mental health well-being of adolescent girls aged 15–19. Participants included adolescent girls in various categories: pregnant, postpartum, living with HIV/AIDS or not, recruited from three youth-friendly primary healthcare facilities using purposive and snowballing sampling methods. A total of 17 adolescents, four healthcare providers, and four parents were interviewed, and a focus group was held, involving six sub-district healthcare program managers.

**Results:**

The factors contributing to mental health among the adolescents were broadly categorised under five themes: 1) Navigating the impact of unintended pregnancy, 2) Negotiating the home environment and other relationships, 3) Barriers around access to services at the facility level, 4) Community healthcare services, and 5) Improving mental health services.

**Conclusion:**

This study explored factors that contribute to or hinder the mental well-being of adolescent girls and the barriers to accessing mental health services. Designing tailored approaches to the identified factors and systemic challenges that counter mental distress for this age group can significantly mitigate the impact on their mental health.

## Introduction

Mental health is increasingly recognized as a critical component of overall well-being [1]. Yet, disparities in access to mental healthcare persist globally [2]. Marginalised populations, including adolescents, experience heightened barriers to mental health services due to systemic inequities, stigma, and socioeconomic challenges [3]. Globally, one in seven adolescents between the ages of 10 and 19 experiences a mental disorder; however, their access to optimal mental healthcare services is inadequate [3]. In sub-Saharan Africa, adolescents face inequitable access to adequate mental health services compounded by poverty, limited healthcare infrastructure, bullying in schools exacerbated by social media, family dysfunctions, difficult home environments, and unsafe neighbourhoods [2,4–6].

In Southern Africa, adolescent girls who are pregnant and/or living with HIV/AIDS represent one of the most vulnerable and underserved groups [7]. Studies highlight that South Africa, with one of the highest HIV prevalence rates globally, faces a teenage pregnancy rate of approximately 16% and a significant burden of mental health conditions among adolescent girls and young women [2,8]. Mental health issues, including depression and anxiety, are disproportionately prevalent among pregnant adolescent girls, particularly those living with HIV due to intersections of stigma, social exclusion, and health challenges [9,10]. Despite their high burden, adolescents often continue to experience barriers to sufficient mental healthcare services and facilities [11].

In South Africa, mental health services for pregnant adolescent girls are critically inadequate, and services are underfunded and under-resourced, limiting their access to vulnerable adolescents [2,5]. Ensuring equity in mental healthcare access for this vulnerable group living with HIV/AIDS is crucial, aligning with global commitments to achieving universal health coverage and the Sustainable Development Goals (SDGs), particularly those aimed at reducing health disparities [12]. This article seeks to explore mental health challenges experienced by adolescents and to understand their access to mental health services within the broader South African sexual and reproductive health services context. These findings aim to address a knowledge gap on adolescent mental health and their access to mental health services, based on insights shared by teenagers, caregivers, and healthcare providers.

### The socio-ecological model

Various frameworks have been used to conceptualise access to healthcare, including patient-centered approaches [13]. The socio-ecological model developed by Bronfenbrenner in the late 1970s [14] provides a comprehensive framework for understanding the complex factors affecting healthcare. This framework conceptualizes individuals as nested within multiple levels of influence, including intrapersonal, interpersonal, organizational, community, and policy levels. The model assumes that immediate surroundings factors have the strongest influence on an individual’s health outcomes, and these include factors such as behaviour, knowledge, attitude, past experiences with health services, socioeconomic status, and beliefs. The interpersonal circle contains factors that focus on immediate interactions and relationships the individual has. These could be relationships with other family members, relationships with peers in schools, and interactions within their neighbourhoods [14]. Organisational factors include the availability of youth-friendly services, opening hours, staff attitudes, availability of medical products such as contraceptives, and services that are offered by healthcare facilities. Community factors include actions and reactions that take part in communities and affect the welfare of the individuals. These may include social stigma, community norms among groups, or organisations that influence adolescent health behaviour. Policy-level factors are influences of policy that affect the community, organisational, interpersonal, and individual-level factors. These are policies that respond to adolescent health needs [14,15].

**Fig 1:**
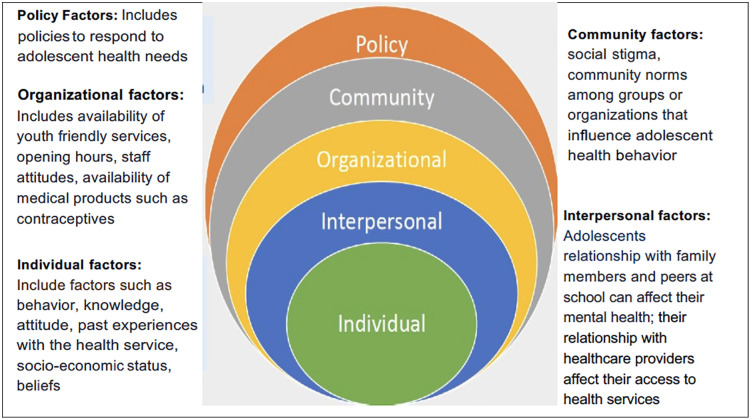
An ecological model for health promotion. Source: Adopted from McLeroy et al. [14].

The SEM has been extensively adopted by various researchers to explain various phenomena. We hypothesize that adolescent mental health and access to sexual and reproductive care services are impacted by various factors and at different levels, as depicted in Fig 1. This model had been adopted from an ecological model for health promotion by McLeroy et al. [14].

As the SEM states that “health is affected by the interaction between the characteristics of the individual, the community, and the environment that includes the physical, social, and political components” [16], the model assumes that health is influenced by multiple factors at various levels [14]. Health outcomes cannot be affected by a single factor, and there is an interlink between factors across the different levels. With such a cross-association of factors, effective interventions should influence factors across multiple levels.

The study used this framework to identify key factors that may influence the mental health and well-being of adolescent girls, which is a fundamental gap in the literature, as well as their access to health services in the City of Cape Town. The model categorizes the factors affecting adolescent mental health across multiple levels, emphasizing both the severity of the challenge and the level of effort required to address it at each stage. Clearly defining the problem at each level enables the development of appropriately tailored strategies and solutions.

## Materials and methods

### Study design

This is an exploratory study that used a qualitative approach. This design was appropriate given the nature of the study: to attain an in-depth understanding of participants’ lived experiences and perceptions of factors related to mental health, HIV, and teenage pregnancy that are not easily quantifiable. The qualitative approach enabled the researchers to explore these factors that are best understood through narratives of participants.

### Study population

Participants for the study included adolescent girls aged 15–19 years in the City of Cape Town (henceforth referred to as Cape Town). The adolescent girls comprised different categories: those who were pregnant, those who were postpartum, those who had never been pregnant, those living with HIV/AIDS, those who were not living with HIV/AIDS, as well as those who had overlapping characteristics, i.e., pregnant or postpartum and living with HIV/AIDS or not. In addition, healthcare providers (nurses) who worked in youth-friendly clinics and parents/caregivers of adolescent girls were selected to participate in the study. Finally, a focus group discussion was held with a purposively selected sub-district team, which included sub-district healthcare program managers.

### Sampling

Purposive sampling was used to select adolescent girls who met the following study inclusion criteria:

1) Between the ages of 15 and 19 years
2) Pregnant and not living with HIV/AIDS
3) Pregnant and living with HIV/AIDS
4) Not pregnant and not living with HIV/AIDS
5) Postpartum and living with HIV/AIDS
6) Postpartum and not living with HIV/AIDS

This criterion was used to gather different life stories and capture narratives across different groups of adolescents with the intent to capture a comprehensive overview of mental health challenges amongst adolescents. Therefore, narratives from participants who were not pregnant and not living with HIV ensured that the findings reflect a broad and balanced perspective that is not skewed or limited to specific subgroups. Although narratives were gathered from adolescents with varying characteristics, this study is not a comparative study. It only aims to attain a comprehensive overview of factors around mental health, pregnancy, and HIV, and how adolescent girls are affected differently. Adolescents were identified through Primary Healthcare (PHC) facilities in Cape Town after obtaining approval from the Western Cape Provincial Health Research Committee and permission from the PHC sub-district healthcare program managers. Permission was granted for 5 selected youth-friendly health facilities. However, adolescents were recruited from only three facilities based on the availability and the willingness of eligible participants to participate in the study. The interviewer recruited adolescents attending various services in the health facilities. Healthcare providers assisted with the identification of eligible participants, who were then approached by the interviewer in the waiting room. Those willing to be interviewed were privately interviewed within the health facility.

Snowballing was also used to identify additional adolescent participants in the study. Interviewed participants were asked if they knew other adolescents who met the inclusion criteria. These participants were asked to share the information sheet with peers. Those who expressed interest in participating were then contacted directly by the interviewer. During this contact, the interviewer explained the purpose of the study, the procedures involved, and issues related to confidentiality and voluntary participation. Willingness to participate was confirmed, and informed consent was obtained before any interviews were conducted. This approach ensured that no information of potential participants was shared without their consent, in compliance with the Protection of Personal Information Act (POPIA). Parents/caregivers of the adolescents were requested for interviews, and those willing to be interviewed were contacted telephonically. Parents who wanted to have telephonic interviews were asked explicitly before the interview started whether they consented to participating in the study. If they agreed, an arrangement was made to post the consent form to them to sign, and a follow-up call was arranged to interview them once they had received and signed the consent form. After the interview, an arrangement was made to send back the signed consent form to the interviewer. Attempts were made to interview partners of the pregnant and postpartum adolescents; however, all of them declined to participate in the study. Healthcare providers (nurses) involved in providing youth-friendly services at the identified health facilities were purposively selected for interviews.

There were 17 interviews that were held with adolescent girls. Since there is no stipulated minimum number of participants with qualitative data, this study intended to conduct 20 interviews with adolescents to attain comprehensive data on their sexual and reproductive well-being, HIV, and mental health. However, due to data saturation observed by the researchers when no new themes or insights were emerging from subsequent interviews, interviews were discontinued after the 17^th^ adolescent participant. In addition to the interviews with adolescents, four interviews were conducted with parents, and four more with healthcare providers. All parents of adolescents were to be interviewed; however, only four were willing to participate. The four healthcare providers were purposefully selected based on their roles as youth-friendly clinic healthcare providers (nurses) from the clinics where the interviews were conducted. Additionally, six sub-district healthcare program managers participated in a focus group discussion to provide further insights on adolescent health on policy-level information related to mental health, HIV, and teenage pregnancy.

### Ethical considerations

Research ethical clearance was obtained from the Biomedical Research Ethics Committee at the University of the Western Cape (Ethics Reference Number: BM19/8/18). Approval to conduct the research was also secured from the Western Cape Provincial Health Research Committee (Reference: WC_202003_003). Permission was obtained from the relevant authorities at the facility and the youth organization levels. Since individuals under 18 years are considered minors in South Africa, parental or caregiver consent was obtained before seeking individual assent from each adolescent participant. Participants were assured that their data would not be shared with their parents or caregivers. Consent was obtained from those 18+ years. To protect confidentiality, participants were assigned study identification numbers rather than using their names. The interviewer went through the information sheet with each participant, providing a detailed description of the aim of the study to prevent any fear of participation amongst participants.

### Data collection process

The interviewer was a qualitative male researcher who holds a Master’s degree. He also had advanced training in qualitative research methods with substantial experience in conducting and analysing qualitative studies, including in-depth interviews and focus group discussions, within health and community-based settings. Their previous work has involved designing qualitative data collection tools, facilitating interviews and group discussions, and applying thematic analysis. Importantly, he also had substantial prior experience conducting qualitative research with adolescents, including adolescent girls, in the same communities. He was therefore selected for his established rapport-building skills, training in ethical and sensitive interviewing techniques, and familiarity with safeguarding procedures. All interviews were conducted in safe, private settings, and participants were informed that they could decline to answer any question or withdraw at any time. These measures helped to mitigate potential discomfort associated with gender dynamics. The interviewer visited the health facility and introduced the research topic to the sub-district healthcare program managers, who then delegated one healthcare provider to assist with identifying prospective participants. Eligible participants were approached and introduced to the study objectives and were invited to participate in the study. It should be noted that the interviewer was the one who explained the research to the adolescents, and not the healthcare providers. It was made clear to all participants that participation was voluntary, and they were free not to participate if they were not willing. All information in the information sheet and the consent form was explained to the participants. Only those who agreed to the study and had signed the consent form were interviewed. Arrangements were made for a private interview in a room at the clinic in a language that each participant was comfortable with. All data collection tools, information sheets, and consent forms were in English and Xhosa, languages that are most understood by participants. Participants were free to make use of or request clarity from the interviewer in either language they were comfortable with.

The interviews comprised narratives of the adolescents’ childhood experiences in general and their experiences with being pregnant, postpartum, living with HIV/AIDS, their family environment, and how these circumstances affected their mental well-being. The adolescents were asked about their experiences regarding access to health services when seeking sexual and reproductive health services at the health facilities and their awareness of mental health policies.

Data was collected between July and October 2021 using a semi-structured interview guide developed by the researchers based on mental health, HIV, and teenage pregnancy. These questions were framed around the Socio-Ecological Model. The interview guide was pretested on three adolescents in the clinics where interviews were to be conducted, and the interview guide was refined based on responses given. Questions around food and nutrition, as well as physical activities among adolescents, were removed as they did not add much value to the research question. Additionally, explicit questions on coping strategies of mental health, HIV, and teenage pregnancy were added to the interview guide as certain responses emerged around these issues. The results from the pretested interviews were not included in the study.

Interviews and a Focus Group Discussion (FGD) were conducted with participants from 3youth-friendly clinics in Cape Town. The interviews ranged between 30 minutes and an hour. The interviewer guided the participants through the questions, providing clarity when needed. Semi-structured questions were used for data collection to explore participants’ narratives around key research themes identified by the research team through the Socio-Ecological Model. An interview guide was developed based on key themes that were relevant to the study. The guide ensured that prompts and follow-up questions were used to gain deeper insights into these key thematic areas. Questions were around the life history of adolescents, their relationship with other people, events outside the home environment, questions around pregnancy, HIV, and mental health. Adolescents’ parents and caregivers were also asked questions about their care of adolescents and any coping strategies they might have given to their children to cope with mental health, HIV, and pregnancy. Healthcare providers were asked questions about sexual and reproductive healthcare services provided to adolescents at healthcare facilities. Questions also pertained to causes of pregnancy and mental health, and how adolescents are coping with these health issues. Sub-district healthcare program managers were asked questions about mental health policy interventions for adolescents. They also discussed factors that caused mental health issues at the community level. The interviewer followed up on any unclear responses to acquire deeper insights into issues around key thematic areas.

A FGD was conducted among sub-district healthcare program managers only to explore shared experiences, consensus, and differing viewpoints on certain institutional aspects of sexual and reproductive healthcare and the mental well-being of adolescents. This lasted for close to one hour. Other groups of participants were not included in the FGD as it was challenging to coordinate and gather them all in one place. No interviews were repeated, and interviews were audio recorded with the participants’ permission. Field notes were made, particularly on respondents’ actions and reactions during the interview process, and these were included in the data transcripts. To avoid redundancy, the concept of data saturation was discussed between two of the researchers (who conceptualised the study and managed data collection) and the interviewer. Training was then provided to the interviewer on how to detect data saturation in order to discontinue data collection once this point had been reached. No data transcripts were returned to participants, as the researchers did not want to reignite past trauma reported by some of the adolescent girls. To maintain consistency across all participants, transcripts were therefore not returned. The interviewer highlighted the availability of a professional psychologist for any participants who may require psychological therapy for past traumas. However, in this particular study, no participant utilised this service.

### Data analysis

A thematic analysis approach was used to analyse data [14]. A deductive thematic approach was used based on the socio-ecological framework presented in Fig 1. First, all the interview recordings were transcribed verbatim by the interviewer. Several interviews that were conducted in Xhosa were also transcribed verbatim and later translated into English. The interviewer went through all interviews and transcripts, ensuring that no information was lost in translation. The transcripts were read several times by all four researchers to identify key issues and were coded. The researchers discussed the identified codes. Themes and subthemes were identified and placed under each of the SEM domains as presented in the SEM. To ensure the objectivity of the data, four researchers (HT, TS, DC, and ML) coded the data separately, discussed the identified codes, and reached a consensus. Triangulation of data was achieved by using different data sources, which included interviews with adolescents, parents, and healthcare providers, to enhance rigor in the study. Researchers ensured reflexivity when analysing data, consistently acknowledging and reflecting on how they engaged with the data to ensure that their background, assumptions, and values did not bias the interpretation of results. Additionally, all researchers went through the interpretation of results to ensure objectivity.

## Results

### Demographic characteristics of participants

Table 1 below summarises the demographic characteristics of the participants involved in the study, providing specific details for the adolescent girls.

**Table 1:** Demographic characteristics of participants.

Variables	Category	Number
Different groups of participants	Adolescent girls	17
	Parents	4
	Healthcare providers (nurses)	4
	Sub-district healthcare program managers	6
Ages of adolescent girls	15 years	3
	16 years	4
	17 years	2
	18 years	5
	19 years	3
Adolescent girls	Currently pregnant	5
	Postpartum	2
	Not pregnant	10
Adolescent girls’ HIV/AIDS status	Living with HIV/AIDS	6
	Not living with HIV/AIDS	11

Five broad themes emerged from the data analysis, as presented in Table 2. These include: 1) navigating the impact of unintended pregnancy, 2) negotiating the home environment and other relationships, 3) barriers to accessing services at the facility level, 4) social challenges, and 5) barriers to accessing mental health services. Using Bronfenbrenner’s socio-ecological model, themes were categorised into: individual, interpersonal, organisational, community, and policy-level domains. A summary of these themes and their corresponding subthemes is presented in Table 2.

**Table 2:** Summary of sub-themes under each domain of the SEM.

SEM domain	Main theme	Sub-themes
Individual factors	Navigating the impact of unintended pregnancy	• The double burden of pregnancy and living with HIV/AIDS • Fears of disclosing pregnancy • Options after pregnancy
Interpersonal factors	Negotiating the home environment	• Support from family/significant other • Dysfunctional families • Financial hardships • Domestic violence and abuse
	Navigating other relationships	• School peer relationships: • Peer pressure • Bullying • Stigma around teenage pregnancy • Unsafe relationships
**SEM domain**	**Main theme**	**Sub-themes**
Organisational factors	Barriers to accessing services at the facility level	• Contraceptive services • Judgement and attitudes of healthcare providers • Unawareness of mental health policies by youth service providers • Inadequate healthcare facilities for adolescents • Healthcare professional shortages
Community factors	Social challenges	• Social stigma around teenage pregnancy • Substance abuse • Unsafe communities
Policy-level factors	Barriers to accessing mental health services	• Lack of mental health guidelines for adolescents

Table 2 summarizes the key themes and subthemes/factors that were identified under each level of the socio-ecological model (SEM).

### Individual factors

a) Navigating the impact of unintended pregnancy

When some adolescent girls discovered that they were pregnant, they experienced significant mental and emotional distress, which was also associated with feelings of isolation and vulnerability. Some realised that they were pregnant and living with HIV/AIDS at the same time. This double burden of unintended pregnancy and living with HIV/AIDS felt overwhelming. Awareness of different options regarding their sexual and reproductive healthcare after pregnancy was burdensome, and their mental and emotional capability to disclose this information was challenging.

i) The double “burden” of pregnancy and living with HIV/AIDS

Fear of disclosing pregnancy as well as living with HIV/AIDS to close friends and family led to mental health distress among adolescent girls. In some instances, adolescent girls unknowingly became pregnant and concurrently contracted HIV, which intensified the impact of the unexpected pregnancy. According to a Healthcare Provider One:

*It becomes very difficult for the newly diagnosed, and it is different compared to those who have been on treatment already, because those who are on treatment… continue with their treatment. For the newly diagnosed, maybe … she was shocked even by the fact that she is pregnant. It happens that one was just coming for family planning or came to remove the implant. It happens sometimes that the implant expired, [and] then she became pregnant. While she received the news of pregnancy, she also received the news of being HIV-positive. So, you can see that she is facing two things at once.* [NOL_PRO001; Healthcare Provider One]

One of the adolescent girls highlighted the following when she was shocked to discover that she was pregnant and also living with HIV/AIDS at the same time:

*I only found out that I am HIV positive when I came here to the clinic, and I found that I was pregnant… Things started to be hectic in my life from that moment, and I could see that depression was also playing with me… I always kept quiet… I was no[t] … eating food that much and I lost weight. I would always close myself inside the house and kept on crying all the time.* [SB006; Pregnant and living with HIV/AIDS]

For this adolescent girl, such a double burden of major health challenges simultaneously resulted in shock, emotional distress, and signs of depression, leading to mental unease.

ii) Fear of disclosing pregnancy

News of unplanned pregnancy and, for some, a new diagnosis of HIV, led to distress. For others, merely the news of an unplanned pregnancy was a shock, leading to psychological distress.

*I did not notice it by myself, but there were people [who] would make comments that “This one should be pregnant, why [is] she becoming fat?” and I would respond to them and say “No, I’m not pregnant”…I thought of buying a pregnancy test. I…did the test on my own. That’s when I discovered that I was pregnant… I was so fearful. I was afraid to disclose it at home.* [NOL008; Postpartum and not living with HIV/AIDS]

A participant, pregnant but not living with HIV/AIDS, said she was too afraid to notify her parents of her pregnancy and left them to notice this. Such thoughts emanated from fears of known or anticipated negative family reactions towards news of the unplanned pregnancy:

“*I was afraid [of] the fact that I disappointed my mother, so I was afraid to talk about it at home”* [NOL007; Postpartum and not living with HIV/AIDS].

Another adolescent girl mentioned:

*I was afraid and was very shocked* [to discover I was pregnant] … *afraid of what my parents would say... I thought that they would shout at me now that I had become pregnant.* [NOL002; Pregnant and not living with HIV/AIDS]

iii) Options after pregnancy

Some adolescent girls were unsure of what actions to take after realising that they were pregnant. Due to fear of disclosing pregnancy to parents as well as facing stigma from peers, some of these adolescent girls had decided to terminate the pregnancy. However, some family members opposed the idea.

*When this child was told that she was pregnant, she decided that she was going to have an abortion … I screamed … ‘There are ten commandments that were written by God that you must not kill. Therefore, when you do the abortion, you are killing.’* [W4P002; Mother of Postpartum adolescent girl, adolescent not living with HIV/AIDS]

This adolescent girl opted to keep the pregnancy but have the baby adopted after birth. However, her parent also objected to this, agreeing to care for the baby:

*… if you take this baby and give [it] to someone we don’t know, when the birthday of your child comes... you will ask yourself questions as to what you have done. And all this will result [in] depression. So, I ask you to... accept that now you have a baby, but this will be mine and not yours.* [W4P002; Mother of Postpartum adolescent girl, adolescent not living with HIV/AIDS]

The findings indicated that certain options that may be taken after pregnancy can create psychological burdens for some adolescent girls, creating an association between pregnancy and mental health.

### Interpersonal factors

b.1.) Relationships in the home environment

Adolescents’ relationships with family members impact both their mental health and access to healthcare. Several factors, such as a lack of family support, having a dysfunctional family, financial hardships at home, and the presence of domestic violence and abuse, created a difficult environment for some adolescent girls, which likely led to mental distress among them.

i) Support from family/significant other

Receiving family support was highlighted by several participants as a factor that would carry them through difficult moments in life. Unfortunately, the families of some adolescent girls did not support unintended pregnancies:

*I did not know that I was pregnant … my mother noticed... She did not say anything at that time, she waited for me to go to school... When I came back from school, she shouted at me and she called some other family members. Everybody was shouting at me... They tried to beat me... At the time, I thought that no one wanted me in life because I am [like] an orphan … I tried to commit suicide* [SB005; Postpartum and living with HIV/AIDS].

The extreme action of wishing to commit suicide highlights the significant mental distress the adolescent girl experienced from the family’s negative reaction towards the unintended pregnancy.

Other families of adolescent girls were supportive. One participant, who was pregnant but not living with HIV/AIDS, had battled with childhood trauma due to her father’s passing away when she was young. This participant was not living with her biological mother (who struggled with alcohol abuse). Her life challenges caused significant mental health distress. Fortunately, the participant’s stepmother, whom she lived with, provided significant emotional support to her through words of encouragement:

*My stepmother is always there and encourage[s] me all the time… She told me that I should forget about things that happened in the past and always focus on the things in my school [school work].* [NOL002; Pregnant and not living with HIV/AIDS]

The encouraging words brought happiness to the participant, leading to a positive mental health impact. Another participant received support towards the pregnancy from her significant other.

*I… informed the father of the child... about my pregnancy, and… he said he does not have a problem. He…supports me most of the time by buying a few items for me, and I feel well. I did not have stress about what my child was going to wear because the father of the child was encouraging me and saying that he was going to take care of [the] child.* [SB006; Postpartum and not living with HIV/AIDS]

Another participant also faced childhood trauma, losing her father to death at a very early life stage: “*The passing away of my father... it was very painful to me…I was thinking that I should just follow him as well* [SB005; Not pregnant and living with HIV/AIDS]*”.* The participant received support and comforting words from relatives: *“My grandmother and grandfather were comforting me. They made me not to always think about my father’s death”.*

ii) Dysfunctional family

Dysfunctional family setup was a contributing factor to mental health distress among adolescents during their formative years. One adolescent participant described the challenges and adverse conditions in her home environment:

*My life was difficult because my mother left me when I was still young …My mother was addicted [to] drinking alcohol … and she dumped me to my father. My father was not working during those times…[he] got some piece jobs as the time went by. From there, he decided to send me to his sister in rural areas.* [NOL009; Pregnant and not living with HIV/AIDS]

A difficult home environment, marked by feelings of early abandonment by a mother at a time when her presence was most needed, exposure to the mother’s alcohol addiction, fathers dying when they were young, and placement with other guardians was likely to contribute to the participants’ distress.

iii) Financial hardships

In some cases, financial hardships within families were significant contributing factors to mental distress. One participant, an adolescent who was neither pregnant nor HIV-positive, explained that life was fine until her father’s injuries disrupted the household income, resulting in considerable economic challenges:

*Life was all right, but my father got shot when I was in Grade 7...He was doing security work…We were living rightfully except [for] this thing that happened … my father went to stay in the hospital for a long time…It was very difficult because he was the only person who was working at home.* [NOL009; Not pregnant and not living with HIV/AIDS].

iv) Domestic violence and abuse

Domestic violence and abuse were critical contributors to an adverse home environment, which, in turn, precipitated mental distress among adolescents. These harmed the home environment and impaired participants’ ability to concentrate on essential aspects such as education. These lead to considerable psychological stress among adolescent girls. “*I could not study well at home because there was always [a] lot of noise, people drinking, and constant fights. So, I could not do anything.”* [SB005; Postpartum and living with HIV/AIDS]

A participant, who experienced challenges in school and had recently failed, highlighted the negative effects of poor conditions at home:

*I am trying to focus, but my family members are always in conflict among each other... They drink almost every weekend, and they open [play] loud music and I cannot find time to study my books well in that environment.* [Not pregnant and not living with HIV/AIDS]

Persistent domestic conflicts and alcohol consumption, which sometimes escalated to physical violence, along with the playing of loud music in the home without regard for the needs of school-going adolescents, emerged as significant disruptive factors to educational progress and mental distress.

b.2.) Relationships with peers from school

Other interpersonal relations, such as peer pressure, bullying, and stigma around teenage pregnancy in schools, were also associated with psychological distress among adolescent girls.

i) Peer pressure

Peer pressure and social comparisons were significant contributing factors to mental health challenges among adolescent girls. A healthcare provider explained:

*I am also surrounded by teenagers in my house… we cannot run away from peer pressure as a contributing factor* [to mental distress]*. The more you want to compare yourself to someone else, the more stressful the situation becomes. A person ends up depressed because she wants something that another person has, while on her side, she cannot afford.* [NOL_PRO002; Healthcare provider Two]

Peer pressure could lead to feelings of inadequacy, causing low self-esteem and anxiety.

ii) Bullying

Bullying was another factor likely to result in mental distress among adolescents. Bullying was described as cyberbullying (internet bullying through social platforms) or in-person bullying at schools. One of the sub-district healthcare program managers recounted:

*…I think adolescents face different challenges than what adults face. I heard that … adolescents … might be bullied and they might be stressed, they don’t want to go to school because they were bullied. So that also plays a… part in their mental health, in their development. I mean you have heard on the news how many suicides there have been because of bullying, because of teenagers and social media and the pressures they face. Now I think adolescents have different stressing things that adults don’t face like social media bullying and school bullying.* [Sub-district Healthcare Program Manager One; FGD]

Sadly, in some cases, bullying, if not resolved timeously and appropriately, might result in extreme incidents, such as suicide among adolescents.

iii) Stigma around teenage pregnancy in schools

A further theme that emerged was participants being stigmatized by school peers for being pregnant. Some pregnant adolescent girls experienced gossip and, at times, mockery and remarks passed around by peers in the school environment:

*… they started gossip[ing] now that they knew about my pregnancy … they gossip whenever they talk among themselves. But they do not come to me... Sometime[s] whenever there is someone who gets* [s] *drowsy in class, they will always make remarks that “There is someone pregnant among us”. … “They make me feel sad*” [NOL002; Pregnant and not living with HIV/AIDS]

Gossip and stigmatization from peers caused deep pain and distress to the pregnant adolescent, and she began to cry while narrating her experience, an indication that peer stigmatisation can be a powerful factor negatively impacting mental health and well-being.

### Organisational factors

c) Barriers to access to services at the facility level
  i) Contraceptive services

Adolescents recounted receiving information about contraceptives from various sources. Clinics, schools, and youth centres were some of the facilities that provided information about contraceptives.

*I found it from the clinic, which was … in the village. I started using them in 2018 because our school is close to the clinic. There, … nurses [would] come out from the clinic and call us on the school lines and talk about the family planning methods.* [NOL004; Postpartum and not living with HIV/AIDS]

For other adolescents, the information about contraceptives was acquired from friends and family.

*The information about family planning methods, I found it from my friend who[m] I trust. I started telling her about things that were happening … [at] home, and then she said, “My friend, you must go to the clinic to protect yourself, as you know how the situation is, people arrive in your home drunk, and some of them keep on touching you, and I don’t know what will happen. So it is better for you to protect yourself”.* [SB005; Postpartum and living with HIV/AIDS]

A healthcare provider shared her views and observations concerning contraceptive information and awareness among adolescent girls:

*Adolescents who come here for the first time [are] mostly accompanied by their parents. If …not,…when they come here, they come with the information that their parents have already told them about which method they should choose. We also play our role in explaining to them about different methods that are available so that they can be able to decide on their own, as they don’t normally need parental consent. You would explain to them about the Nuristerate for the two months or Petogen, which is taken for 3 months. There is also an implant for three years and the IUD for 5 years. They also have their own choice to make… at least they must know a variety of methods that are available so that one cannot just only choose the method that the mother has recommended.* [NOL_PRO001; Healthcare Provider One]

Healthcare providers play a pivotal role in sharing information about the different family planning methods with adolescents, increasing their knowledge and awareness of contraceptives.

However, some misinformation and misconceptions on contraceptive use became a barrier to using some of these methods of contraception, exposing adolescent girls to the risk of pregnancy and STIs:

*I guess I was scared as I heard some negative stories about them [contraceptives]… One of my friends who was in class with me said, … “I think the injectable makes you fat”* [NOL007; Pregnant and not living with HIV/AIDS]

A healthcare provider, too, highlighted perceived myths around the use of contraceptive methods.

*Sometimes they come with myths that they hear from their communities about what family planning does. For example, if one uses family planning, she may end up not being able to conceive in future. Maybe someone comes already with the information from her mother in a way that she must take the Nuristerate because Petogen is for older people. You would find that when they come here, that information already sits in a child’s mind. Again, they [are] present[ed] with a myth that family planning disturbs their periods [menstruation] process in a way that they become irregular. As a result, you noticed that some of them end up stopping taking or using family planning.* [NOL_PRO001; Healthcare Provider One]

Increasing the awareness and the sources of correct information about contraceptives can assist adolescent girls in making better-informed decisions about their sexual and reproductive healthcare.

ii) Judgement from healthcare providers

Another element mentioned by one of the healthcare providers impeding adolescent access to some healthcare services was judgment by healthcare providers, which should cease:

*I feel like there is still a lot more that needs to be done because other diseases come up and pass, but the stigma that is with HIV is still difficult to remove from other people. So, we* [healthcare providers] *must try to talk to the clients as healthcare providers and not judge a child and say, “No, a child at the age [of] 18, what were you doing?” and things like that. A child must be comfortable to talk with us about everything that she feels …. This will help a lot so that clients will not default.* [NOL_PRO002; Healthcare Provider Two]

A consequence of such judgment from healthcare providers is adolescent resistance to accessing and utilising these services. The same healthcare provider highlighted that some patients living with HIV/AIDS would rather default on their HIV medication and treatment than engage with judgmental healthcare providers, thus acting as a barrier to access to services at the facility level.

iii) Unawareness of mental health policies by youth service providers

Some providers were familiar with HIV guidelines but lacked knowledge of mental health guidelines:

*I don’t know about guidelines at all, child and adolescent mental health policy, currently being rolled out at the provincial level... this is the first time I hear about it … I know only HIV guidelines.* [NOL_PRO001; Healthcare Provider One]

The majority of youth healthcare service providers interviewed were either unaware of mental health policies, including broader child and adolescent policies, or had only heard about them without having read them:

*I am not aware of [the] new adolescent mental health policy. I don’t deal much with mental health issues in depth … I remember that there were earlier policies* [NOL_PRO004; Healthcare Provider Four]

Healthcare providers demonstrated a lack of adequate specific knowledge about mental health guidelines tailored for youth. This knowledge gap poses a significant barrier to their ability to provide adequate mental healthcare services to adolescents experiencing this challenge. Without a comprehensive understanding of these guidelines, the detection, diagnosis, and treatment of mental health conditions among adolescent girls is limited, increasing the exclusion of this group of people from adequate mental health services.

iv) Inadequate healthcare facilities for adolescents

An organisational barrier highlighted by participants as limiting the accessibility of mental health services was the lack of sufficient infrastructure. While the need for such mental health services exists, the available infrastructure providing these services is limited. A sub-district healthcare program manager explained:

*… the infrastructure that we have in primary care is hopelessly inadequate, buildings are too small, we have become comprehensive, and we take on adolescent care and … mental health, we operate in the same building … but the building space does not allow* [for mental health service provision] *…* [Sub-district Healthcare Program Manager Two; FGD]

She continued to emphasise that the limited infrastructure at the healthcare facility compromised the provision of one service (mental health services) at the expense of another (provision of medicine). However, providing medicine to adolescents alone is insufficient; counselling and therapy sessions are needed.

v) Healthcare professional shortages

Having a shortage of healthcare professionals was a further organisational barrier limiting adolescents’ access to mental health services. There are too few professional psychologists available to provide adequate counselling sessions:

*We have been introduced to the psychologists. I just can’t remember how many we have in the whole City [of Cape Town]. Is it maybe 4? So how do you introduce 4 people into a service where there is like 50% of people that* [need them] *…Who do you refer to them? Who do you take?* [Sub-district Healthcare Program Manager Two; FGD]

Decision-making on who should receive such counselling from professional psychologists becomes difficult. Inevitably, some adolescents will not receive counselling sessions, increasing their risk of anxiety and depression.

### Community factors

d) Social challenges
  i) Stigma in the community

Similar to stigma in schools, judgment in society around teenage pregnancy has mental health implications. A participant recalled how she was treated differently by her community members for becoming pregnant when she was also living with HIV/AIDS.

*They* [the community members] *did not make me feel happy…During the time I became pregnant, there were some people … judging me that “Yho!* [a mocking expression of surprise] *we knew that she was going to be pregnant and…[she] is not going to continue with school anymore”.* [SB005; Postpartum and living with HIV/AIDS]

Such judgmental attitudes from community members can lead to negative mental health outcomes for participants.

ii) Substance abuse

The abuse of substances such as alcohol, cannabis, and other harmful drugs is heightened as a community hazard for adolescents in communities.

*It has a lot of contribution because some of them* [adolescent girls] *would like to experience everything when they reach [the] teenage stage and they do not want to remain behind... A certain group will be against another group, and there are those fighting at school among those grouping[s] and they use the substance, and a person end[s] up depending on that substance. This all leads to someone among them becoming mentally disturbed.* [NOL_PRO002; Healthcare Provider Two]

Heavy dependency on these substances and conflicts about them within schools can lead to mental health deterioration, resulting in mood disorders, anxiety, and depression among adolescent girls.

iii) Unsafe communities

Lack of safety in communities is an important factor that can lead to fear and anxiety among adolescent girls. Acts of sexual violence, particularly rape of children and women, were major concerns, causing a sense of insecurity in some communities. One of the participants narrated the following regarding the issue of safety within their community:

*Sometimes you’d hear …[about] children who got raped and some other people who are suffering in the communities … People who are raping women [and] children, they must be caught and put in prison so that we can be safe in the communities.* [NOL008; Postpartum and not living with HIV/AIDS]

Living in unsafe communities, which are also a potential site of sexual violence, can lead to significant mental distress, particularly in adolescent girls.

### Policy level issues – provision of mental health services

e) Lack of PACK guidelines for adolescents

Nationally recognised policy guidelines for mental health are important. The Practical Approach to Care Kit (PACK) was developed as a comprehensive set of guidelines with the primary objective of guiding clinical decision-making during patient consultations in South Africa [17]. These guidelines are to ensure that care aligns with policy and delivers integrated, comprehensive primary healthcare.

However, while well-developed versions exist for children under 13 (PACK Child) and adults (PACK Adult), currently there are no finalised PACK guidelines for adolescents. One of the sub-district healthcare program managers described the situation, “*… we don’t have the adolescent PACK guidelines, but it is actually coming soon. So that actually focuses … on adolescents…”,* [Sub-district Healthcare Program Manager Three; FGD].

Lack of specific adolescent PACK guidelines increases the vulnerabilities of adolescents to misdiagnoses and inadequate treatment and care for health conditions, particularly mental health issues.

## Discussion and conclusion

The findings of this study reveal a complex interplay of factors contributing to adolescent mental health challenges, exacerbated by pregnancy and/or HIV/AIDS for some adolescent girls. Influences range from individual, family, organisational, community, and health systems domains. At an individual level, unintended pregnancy, particularly when coupled with living with HIV/AIDS, can lead to mental distress emanating from fear of disclosure, shock, and pervasive stigma around unintended pregnancy. This double burden increases feelings of isolation, anxiety, and depression among adolescent girls. This echoes findings elsewhere that unintended pregnancies disproportionately impact adolescent girls, especially when intersecting with socio-economic vulnerabilities and HIV [7,9,12,18].

Within the interpersonal, family domain, adverse home environments marked by a lack of family support, dysfunctional family dynamics, financial hardships, and domestic violence and abuse emerge as critical determinants of poor mental health. The absence of consistent emotional support and open communication not only deepens feelings of isolation and despair but may also negatively influence future sexual and reproductive decisions. These findings are consistent with evidence that stable, supportive, safe, and conducive home environments are crucial for fostering adolescent resilience against mental distress and other life challenges [9,19]. Bullying at school and elsewhere is highly prevalent and needs to be addressed as a major stressor for adolescents [6]. HIV emerged as an exacerbating factor from one participant’s comments on having already been gossiped about by community members while living with HIV/AIDS before becoming pregnant. This worsened after she became pregnant, leading to much stress. Healthcare providers also commented on the additional shock adolescent girls experience when, in addition to the diagnosis of HIV, they were told they were pregnant. In these cases, the negative feelings were extreme to the extent that one of the adolescents wished to commit suicide. This shows that the burden of falling pregnant and concurrent HIV acquisition can be a big trigger for potential mental health issues. This may have lifelong implications for the adolescents’ mental health. Established relationships in schools and communities have an impact on mental health, and maintaining stable interpersonal relationships can significantly contribute to mental well-being among adolescents.

At an organisational level, certain barriers to health services, including misinformation about contraceptives from peers or their communities, judgment from some healthcare providers, the lack of mental health information from some healthcare professionals, inadequate infrastructure, and a shortage of professional psychologists, can increase adolescents’ vulnerability to mental health issues.

At the community level, the pervasive social stigma surrounding teenage pregnancy significantly contributes to adverse mental health outcomes, exacerbating feelings of shame and low self-esteem [18]. In addition, the prevalence of substance abuse of alcohol, cannabis, and other drugs in these communities is linked to increased rates of mood disorders, anxiety, and depression among adolescent girls [2]. Moreover, living in unsafe communities where incidents of violence (including sexual violence) are common further intensifies feelings of insecurity and anxiety, particularly among adolescent girls. This underscores the urgent need for comprehensive community-based interventions and enhanced legal protections [5,7].

Within the policy level domain, significant gaps exist in the design and delivery of mental health services tailored for adolescents. This is highlighted by the lack of adolescent PACK guidelines. Using adult PACK guidelines for adolescents can lead to misdiagnosis, as mental health issues amongst adults can differ from issues among adolescents. Thus, adult PACK guidelines fail to meet the specific needs of this group, necessitating targeted interventions and specialized training for healthcare providers. Even if adolescent PACK guidelines are developed and implemented, if there is limited awareness of these guidelines and inadequate training of some healthcare providers, challenges to meeting adolescents’ mental health needs will remain [20–22].

The results showed that adolescents who were pregnant/postpartum and infected with HIV experienced more severe mental health issues than other participants (those who were not pregnant and/or not infected with HIV). Lack of family support during pregnancy created a greater adverse home environment, contributing to mental health distress among pregnant adolescents. These findings are supported by other studies, such as Monyasa et al. [23], where they found a higher prevalence of depression among pregnant women relative to non-pregnant adolescents. Similarly, a study in rural South Africa found that pregnant and postpartum adolescent girls experienced various mental health challenges due to stigmatisation, lack of support from family and friends, parenting demands, and poor performance in school [19]. Additionally, a study in Uganda [24] also observed a higher prevalence of psychological distress amongst pregnant young women living with HIV/AIDS relative to non-pregnant women living with HIV/AIDS, findings that are similar and support our findings. Hence, there is a need for comprehensive mental health interventions and other strategies for all adolescent girls, particularly those who are pregnant and/or are infected with HIV.

The findings highlight the urgent need for more comprehensive approaches that simultaneously address individual vulnerabilities, strengthen interpersonal relationships, approaches around social challenges, and resolve organisational and policy-level barriers to mental health services. Interventions tailored for adolescent girls are essential for mitigating the intersectoral factors contributing to mental health challenges that impact adolescent girls and for advancing their access to appropriate healthcare services.
